# Conceptual Spaces of the Immune System

**DOI:** 10.3389/fimmu.2016.00551

**Published:** 2016-12-13

**Authors:** Walter Fierz

**Affiliations:** ^1^Labormedizinisches Zentrum Dr Risch, Schaan, Liechtenstein

**Keywords:** conceptual spaces, immune system as a cognitive system, vaccine development, diagnostic tests, immune therapy

## Abstract

The immune system can be looked at as a cognitive system. This is often done in analogy to the neuro-psychological system. Here, it is demonstrated that the cognitive functions of the immune system can be properly described within a new theory of cognitive science. Gärdenfors’ geometrical framework of conceptual spaces is applied to immune cognition. Basic notions, like quality dimensions, natural properties and concepts, similarities, prototypes, saliences, etc., are related to cognitive phenomena of the immune system. Constraints derived from treating the immune system within a cognitive theory, like Gärdenfors’ conceptual spaces, might well prove to be instrumental for the design of vaccines, immunological diagnostic tests, and immunotherapy.

## Introduction

The mammalian immune system is often described like a cognitive system as exemplified by the following commonly used ways of expressing immune functions:

- Components of the immune system (B-cells, antibodies, T-cells, and MHC molecules) *recognize*^1^ (see) antigens.
- The immune system *responds* to an antigen.
- The immune system *distinguishes* self from non-self.
- The adaptive immune system has the capability to *learn* and has *memory*.

In this way, putative cognitive functions of the immune system have been expressed up to now by loose analogies to cognitive functions of the neuro-psychological system, but an analysis of immune cognition within a theoretical framework of cognitive science has been missing. Consequently, serious discussions of cognitive phenomena in the immune system have always been plagued by the obvious inadequacy of such a metaphoric usage of terms from the neuro-psychological field.

A treatise of both the immune and neuro-psychological cognition on a meta-level within a theoretical framework would allow formulating scientifically sound assertions about cognitive functions of the immune system without falling into the trap of making suggestive but undue shortcuts between the two quite diverse cognitive systems.

The development of mathematical models of the immune system has already led in the direction of abstracting and generalizing immunology as an information processing system (1). More than that, it has inspired computer engineers to develop artificial immune systems (AIS) in the form of software (2) and hardware (3) systems that take advantage of the principles of cognitive immune functions that nature has invented, similar to the artificial neuronal networks (ANN) that are inspired by the neuronal system (4). However, these mathematical models are specifically oriented toward the immune system or toward application in information processing and they do not provide a generic cognitive framework.

Some years ago, Irun R. Cohen proposed a cognitive paradigm in which preformed internal images guide and restrict functions of the immune system (5). These are not only images of infection but also an image of self, which he termed immunological homunculus (6). Obviously, these and others, less radical ideas go far beyond the classical theoretical framework of immunology and directly draw on notions from the field of neuro-psychology. Such parallels are suggestive but lack a concise theoretical model. In this article, some of these somewhat provocative ideas are taken up, and it is examined whether a new theory in cognitive science, Gärdenfors’ framework of conceptual spaces^2^ for modeling representations of information based on geometrical structures, is applicable to cognitive functions of the immune system. In this way, Cohen’s cognitive paradigm could be embedded in a cognitive theory that encompasses also other systems, particularly the neuro-psychological system. An extensive description of the theory can be found in Gärdenfors’ book about conceptual spaces (7). A recent short summary of Gärdenfors’ theory can be found in Ref. (8).

In the following, Gärdenfors’ framework of conceptual spaces will be applied to questions of immune cognition. The basic notions, like quality dimensions, natural properties and concepts, similarities, prototypes, saliences, etc., shall be related to cognitive phenomena of the immune system.

This endeavor has three purposes as follows:

1. To demonstrate the applicability of the theory of conceptual spaces to the context of the immune system as a cognitive system.
2. To demonstrate the value of conceptual spaces in explaining immunological phenomena.
3. To demonstrate the constructive merit of conceptual spaces for vaccine development and design of diagnostic tests and immune therapy.

## Conceptual Spaces

The framework of conceptual spaces builds on geometrical structures. In short, it defines a concept (e.g., apple) as a set of properties (e.g., green, sour) in a number of correlated domains (e.g., color, taste) of integral quality dimensions (e.g., hue/saturation/brightness).

The theory of conceptual spaces is based on the following fundamental notions (7) (see Table 1, for examples):

- A conceptual space is a geometrical structure consisting of a number of quality dimensions sorted into domains.
- Quality dimensions represent various qualities of objects in different domains, for example, temperature, weight, height, width and depth, brightness, wavelength, and luminance. They have specific topological or geometric structures or metrics (e.g., °C, kg, m, nm, and cd/m^2^).
- A domain is a set of integral, non-separable dimensions that are separable from all other dimensions, e.g., color. Integral means that it is not possible to give a value in one dimension without giving a value in the other dimension, e.g., hue and brightness or the pitch of a sound and loudness.
- A property is a region in some domain (e.g., red, sweet). A natural property is a convex region (Criterion P). Criterion P assumes that the notion of betweenness is meaningful for the relevant dimensions. Convex means that for any two points in a region, all points in between the two are also in that region. Properties, as defined by Criterion P, form a special sub-class of concepts. A property is based on a single domain, whereas a concept may be based on several domains.
- A concept is a set of correlated properties with information about how the regions in different domains are correlated (Criterion C). An apple, e.g., is a concept with certain correlated properties in the domains of color, shape, taste, etc. Thus, concepts are not just sums of properties. Criterion C assumes correlations between regions from the different domains associated with the concept. In the example of the apple concept, there is a strong correlation between sweetness in the taste domain and sugar content in the nutrition domain, with a weaker correlation between a red color and a sweet taste.

**Table 1:** **Conceptual spaces in the immune and neuro-psychological systems**.

Immune system		Conceptual spaces	Neuropsychological system	

**Example**		**Concept**	**Example**	
Influenza virus		Set of correlated properties	Apple	

**Property**				
**Regions**	**Domains**	Region in a domain of a conceptual space	**Domains**	**Regions**
Hemagglutinin	Envelope antigen		Color	Red–yellow–green
Polymerase	Non-structural antigen		Taste	Sweet–sour

**Domain**				
**Examples**	**Integrality**	Set of integral dimensions	**Integrality**	**Examples**
Hemagglutinin	Molecule/cluster of molecules		Non-separable qualities	Hue/saturation/brightness

**Quality dimension**				
**Hemagglutinin**		Dimension with a geometrical structure	**Color**	
**Metrics**	**Epitope**		**Quality**	**Metrics**
Binding-affinities	B-/T-cell epitope	**Phenomenal**	Hue/saturation/brightness	Color spindle
Aminoacid sequence	Chemical nature of binding site	**Physical**	Wavelength/luminance	nm cd/m^2^

**Examples**		**Prototype**		**Examples**
	Immunodominant epitope	Geometrical center of a cluster of points in a domain	Typical color	Apple green

**Examples**		**Similarity**		**Examples**
	Shared antigen/epitope leading to cross-reactivity	Decaying function of geometrical distance	Shared property/quality of concepts	Apples and oranges are similar in color and shape

		**Salience**		
Frequency of B-/T-cells reactive to an epitope, immunogenicity, size of memory cell pool		Context-dependent weight of a dimension		

Based on these definitions, other basic notions are introduced, like saliencies (weights), similarities, and prototypes, to explain concept combinations, dynamic concept formation, and learning.

Here, it is conjectured that this framework can successfully be applied for the immune system as follows:

- Epitopes are quality dimensions with affinity as metrics.
- Antigens are natural properties.
- Microbes are natural concepts.

## Epitopes as Quality Dimensions of the Immune System’s Conceptual Space

The notion of a dimension should be understood literally. It is assumed that each of the quality dimensions is endowed with certain geometrical structures (in some cases they are topological or ordering structures) [(7), p. 6].

**Figure 1:**
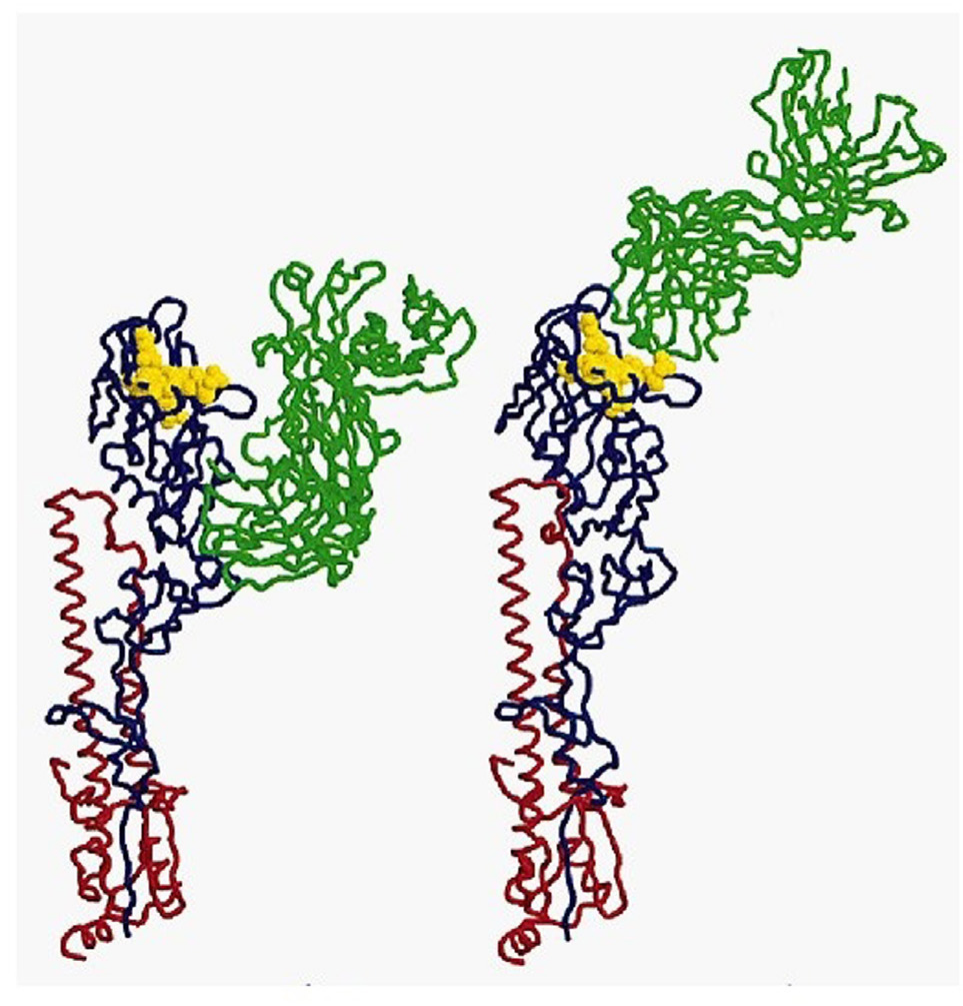
**A complex of influenza hemagglutinin with a neutralizing antibody that binds outside the virus receptor-binding site**. Ribbon diagram of the complex showing one BHA monomer (HA1 in blue, HA2 in red) and the HC45 Fab (in green); the receptor-binding site is shown in yellow; for comparison, the X31 HA–HC19 Fab complex is shown on the right (9). Reprinted by permission from Macmillan Publishers Ltd.: Nature Structural and Molecular Biology (9), copyright (1999).

**Figure 2:**
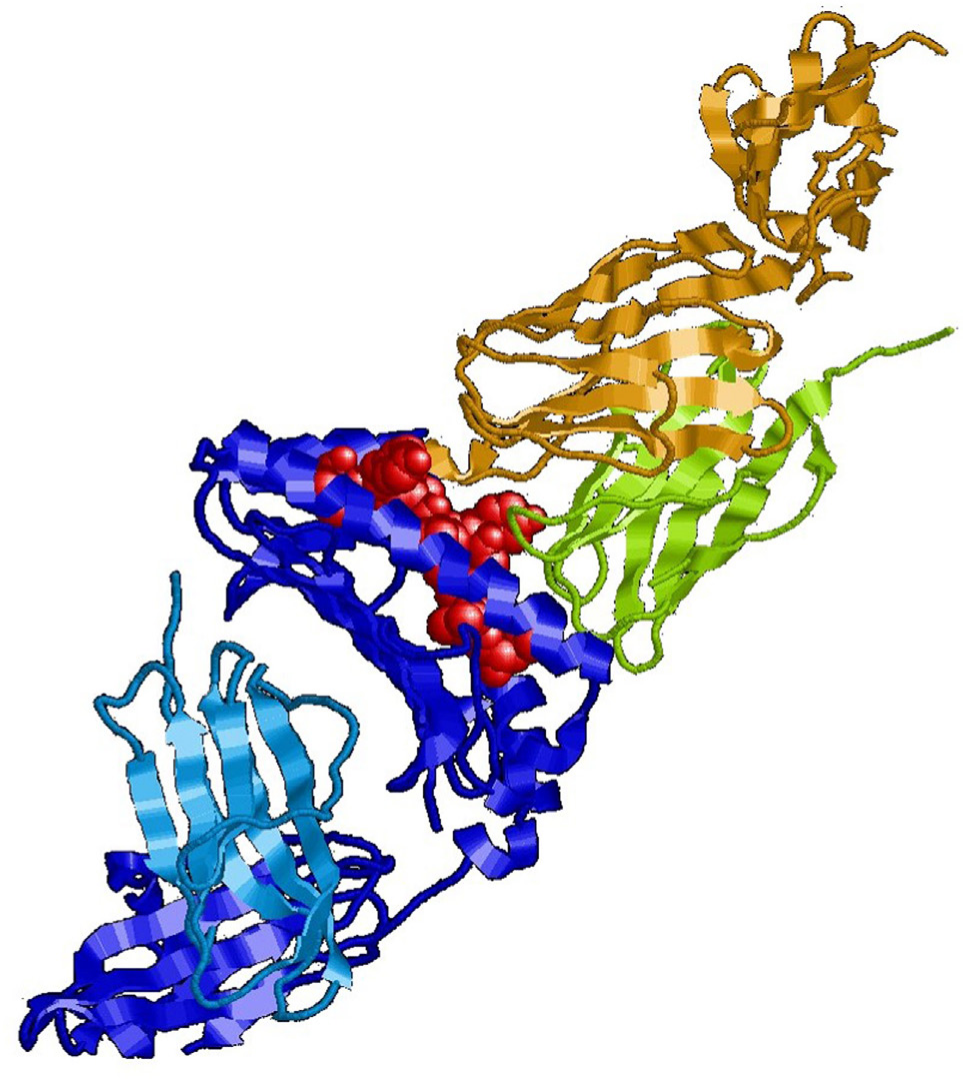
**MHC class I molecule (blue) presenting a peptide (red) to the T-cell receptor (green/olive)**. For review, see Ref. (10).

In immunology, the basic unit of recognition is an epitope of an antigen. Epitopes, also known as antigenic determinants, are bound (recognized) by the complementarity determining region of an antibody (B-cell receptor) (see Figure 1). T-cells, on the other hand, recognize epitopes as peptide fragments, which have been processed by an accessory cell and presented to the T-cell receptor in the cleft of an MHC molecule (see Figure 2).

Here, it is proposed that an epitope can be represented by a quality dimension in the conceptual space of the immune system. The metrics of the epitope as a quality dimension is, however, not immediately obvious. According to Gärdenfors, it is important to distinguish different interpretations of dimensions:

The phenomenal interpretation concerns the cognitive structure (perceptions, memories etc.) of humans or other organisms. The scientific interpretation, on the other hand, treats dimensions as part of a scientific theory [(7), p. 8].

The proposition here is that the metrics of the dimension defined by an epitope is given by the affinity of the receptor binding to the epitope (phenomenal interpretation). Alternatively, epitopes can be defined by the chemical nature and physical shape of the recognized entity: proteins, polysaccharides, lipids, etc. (scientific interpretation). In proteins, primary, secondary, tertiary, or even quaternary structures can serve as epitopes. In this article, the phenomenal interpretation, the view of the immune system, will be used as the metrics of the epitope dimension.

### Comparison with the Shape Space Model of Perelson

**Figure 3:**
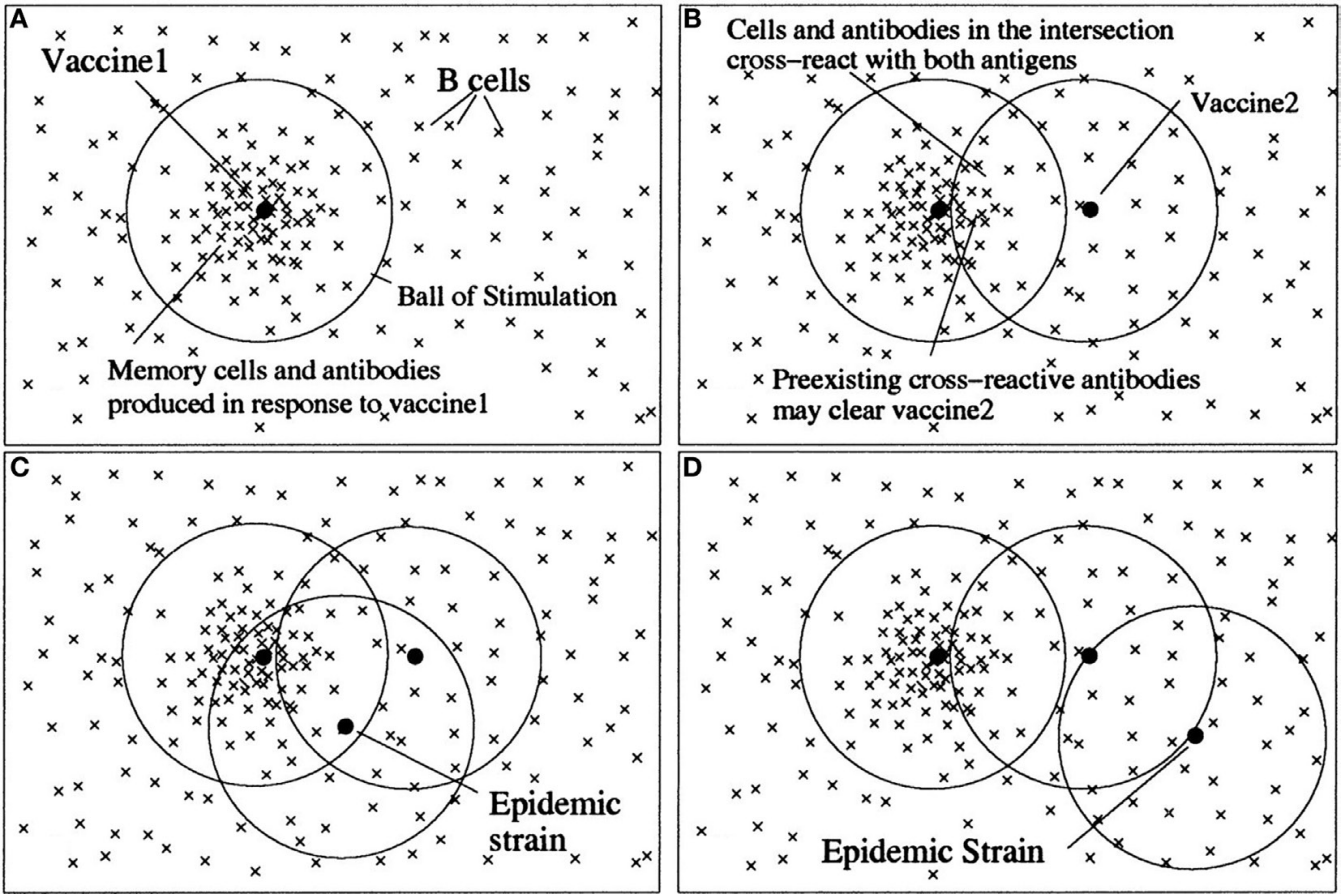
**An illustration of the antigenic distance hypothesis**. *Shape space* diagrams are a way to illustrate the affinities between multiple B cells/antibodies and antigens, and also the antigenic distances between antigens. In these shape space diagrams, the affinity between a B cell or antibody (×) and an antigen (•) is represented by the distance between them. Similarly, the distance between antigens is a measure of how similar they are antigenically. **(A)** B cells with sufficient affinity to be stimulated by an antigen lie within a *ball of stimulation* centered on the antigen. Thus, the first vaccine (vaccine 1) creates a population of memory B cells and antibodies within its ball of stimulation. **(B)** Cross-reactive antigens have intersecting balls of stimulation, and antibodies and B cells in the intersection of their balls – those with affinity for both antigens – are the cross-reactive antibodies and B cells. The antigen in the second vaccine (vaccine 2) will be partially eliminated by preexisting cross-reactive antibodies (depending on the amount of antibody in the intersection), and thus the immune response to vaccine 2 will be reduced. **(C)** If a subsequent epidemic strain is close to vaccine 1, it will be cleared by preexisting antibodies. **(D)** However, if there is no intersection between vaccine 1 and the epidemic strain, there will be few preexisting cross-reactive antibodies to clear the epidemic strain quickly, despite two vaccinations. Note, in the absence of vaccine 1, vaccine 2 would have produced a memory population and antibodies that would have been protective against both the epidemic strains in **(C)** and **(D)**. For an antigen with multiple epitopes (such as influenza), there would be a ball of stimulation for each epitope (12). Copyright (1999) National Academy of Sciences, USA.

The view of epitopes as quality dimensions with affinity as metrics is closely related to Perelson’s shape space model of antibody–antigen interaction (11). There, each antibody and antigen is regarded as a point in an *n*-dimensional shape space, and the affinity between an antigen and antibody is related to the geometrical distance between them. In this way, an *n*-dimensional “ball of stimulation” is defined within which affinity is high enough to cause stimulation of antibody-producing B-cells (Figure 3).

The difference of the space shape model to the view described in this article lies partly in the usage of terms. Here, the basic unit of recognition is the epitope, and the antigen is made up from various epitopes. The term dimension is used here for one antibody–epitope interaction, whereas Perelson models the antibody–antigen complementarity with multiple implicit shape space dimensions, which are not in fact physically defined. Like that, Perelson’s model somehow merges the two separate views of (1) one epitope – one antibody – one affinity dimension and (2) one antigen – multiple antibodies – multiple epitopes – multiple affinity dimensions (see next section).

Irrespective of these differences in terminology, Perelson’s model is instrumental in describing cross-reactivities between two antigens as overlapping regions of two “balls of stimulation.” This “antigenic distance hypothesis” is closely related to the representation of cross-reactivity within the framework of conceptual spaces (see further on).

## Antigens as Natural Properties of the Conceptual Space of the Immune System

A central idea of the theory of conceptual spaces is expressed in Criterion P of Gärdenfors: “A natural property is a convex region of a domain in a conceptual space” [(7) p. 71].

A domain, which plays a pivotal role in the framework of conceptual spaces, is defined as “a set of integral dimensions that are separable from all other dimensions” (p. 26). The key notion in the definition of a domain is the integrality, which makes properties separable from each other.

In immunology, an integral unit of recognition is the antigen. It usually is a single molecule or a structural cluster of molecules, like a viral capsid. An antigen may encompass one or several epitopes, but physically these epitopes are integrated within the molecular structure of the antigen. Under natural conditions epitopes of an antigen are non-separable and B-cells recognize antigens usually in their native form. For recognition by T-cells, on the other hand, antigens are physically disintegrated (processed) in antigen-presenting cells, and small peptides, embedded in the cleft of MHC molecules, are presented to the T-cell receptors (Figure 2). However, since B-cells require help from T-cells, which is delivered over a short distance range, a cooperative B-/T-cell response is dependent on a close integration of epitopes for B- and T-cells. In this way, the structural integration of B- and T-epitopes in an antigen provides the physical grounding of a domain of integral dimensions of epitopes. A further evidence for the assumption that epitopes of an antigen are integral for immune recognition is the observation that epitopes recognized by B-cells can lose their integrity when protein antigens are denatured.

Here, it is proposed that an antigen can be represented by a region in a domain (natural property) of the conceptual space made up from several integral (non-separable) dimensions (epitopes). In other words, the antigenic property of a molecule is geometrically defined by the binding affinities of the B- and T-cells toward the recognized constituent epitopes.

Whether such region in a domain of epitopes is convex is, however, not immediately obvious. Convexity is defined as such:

A subset of C of a conceptual space S is said to be convex if, for all points x and y in C, all points between x and y are also in C [(7), p. 69].

The notion of convexity in the geometrical structure of a domain is based on the notion of betweenness:

Criterion P presumes that the notion of betweenness is meaningful for the relevant domains (p. 71).

In a conceptual space made up from affinity dimensions of epitopes, the notion of betweenness is well defined, and it is reasonable to assume that a region in a domain of several epitope dimensions of an antigen is convex, but this assumption is not a priory granted, albeit amenable to experiments. The significance of convexity of natural properties lies in the fact that it leads to the notion of prototypes, since in a convex region there are ways to define a geometrical center.

### Prototypes

Epitopes are recognized by randomly generated and clonally distributed receptors of the adaptive immune system. Thus, there is a certain variability of how an antigen is seen by the immune system. More than that, a single epitope is recognized by a multitude of clones of receptor-bearing lymphocytes, a normal phenomenon that is called polyclonal immune response. This variability can be expressed by the different affinities of the individual receptor clones recognizing the epitope. Taken together, an immune response to an antigen is made up of several exemplars of responses to epitopes, which can be represented in a conceptual space by several points in a region of a domain. The geometrical center of such a cluster of points can be perceived as a prototype of an immune response to an antigen.

On a population level, among various individuals, particular prototypes again form a cluster of points in a conceptual space. Such population prototypes, as defined, e.g., by international standard sera (i.e., pools of individual immune sera), form the basis for comparing immune responses with diagnostic tests. Furthermore, the design and development of vaccines assume the existence of prototypic immune responses to microbial antigens.

## Microbes as Natural Concepts of the Immune System

According to Criterion C of Gärdenfors’ conceptual space framework,

a natural concept is represented as a set of regions in a number of domains together with an assignment of salience weights to the domains and information about how the regions in different domains are correlated [(7), p. 105].

An important point here is the notion of correlation, leading to the question which antigens as natural properties recognized by the immune system are correlated enough to form a natural concept.

The tenet here is that a microbial entity (virus, bacterium, etc.) is fulfilling this constraint and is *viewed* by the immune system as a natural concept made up of various correlated properties (antigens). The correlation is given by the physical association of the antigens in space and time. The *perspective* taken by the immune system depends on the context within which the microbe is encountered and on the state of learning by the immune system.

Infection by a microbial agent normally leads to a whole set of immune responses against various antigens and antigenic epitopes of the infecting agent. The responses to different antigens of the microbe are usually correlated in time and space. In this way, the immune system develops a concept of the microbe infecting the organism. More than that, the persistence of memory cells of the immune system carries this concept into the future. Re-infection by the same microbe elicits an accelerated and more vigorous response, which in many cases is sufficient to eliminate the intruder before it can establish itself. This leads to the well-known protection imparted by certain childhood infections. Thus, learning and memorizing concepts by the immune system is pivotal for survival, and therefore, it is based on an evolutionary origin. On top of that, vaccination techniques allow exploiting these cognitive functions of the immune system to constructively teach the system new concepts.

The set of immune responses raised to a particular microbe is, however, different between different individuals, depending on the genetic makeup of the individual and the previous immunological experiences. Consequently, concepts of individual immune systems will vary. Overall though, as for antigens (see above), there exist prototypes of immune responses to a particular microbe. Again, such prototype is defined by the center of a cluster of points in the conceptual space of a whole population.

### The Problem of the Concept of Self

A somewhat controversial issue in immunology is the question whether the immune system has also a natural concept of self, or as Cohen has put it, an immunological homunculus (6). It is not the intent of this article to take position in this debate, but rather to point in the direction where the solution might lie. The tenet is that immune cognition of self vs. non-self, or non-infectious-self vs. infectious-non-self (13), or dangerous vs. harmless (14) is a problem of immune semantics (not to be mixed up with semantics of immunological science, see further on). In other words, the conceptual space of the immune system might well have dimensions of self-epitopes and properties defined by domains of self-dimensions (auto-reactivity) or even concepts of self as a whole or of various tissues and organs – but the issue is that only the semantic context, which defines the saliences of self-domains, provides to the immune system the meaning of such recognition of self.

Here, the difference between the phenomenal interpretation and the scientific interpretation comes into play. From the perspective of the immune system, self and non-self is a matter of how an antigen is presented, recognized, and finally reacted to (phenomenal interpretation), irrespective of the provenance of the antigen, i.e., irrespective of its scientific classification as self (belonging to the organism) or non-self (foreign to the organism).

### Similarities – Cross-reactivity and Molecular Mimicry

An important notion in the theory of concepts is that of similarity. In conceptual spaces, similarity is related to shared properties and geometrical distances. The similarity of concepts in immune recognition is defined by shared antigens. When the distance between two regions of a domain is small, antigens (represented by these regions) become indistinguishable for the immune system. Antigens are similar, when they share epitopes (quality dimensions). Recognition of similar antigens shared by different concepts (microbial entities) is well known in immunology as cross-reactivity. The antigenic distance hypothesis formulated by Perelson and Oster (11) and successfully applied to the real world problem of influenza vaccination (12) fits well in this framework. Similarities between antigens are represented in both theories, Perelson’s and Gärdenfors’, by distances in a geometrical space (Figure 3).

When microbes share antigens or epitopes with the host organism, there will be cross-reactivity to self, a phenomenon called molecular mimicry, which is one of the postulated mechanisms leading to autoimmune disease (15).

## Concept Formation and Learning

The formation of a concept by the immune system requires that recognition of different regions of domains (antigens) is correlated. Such correlation is provided in the immune system by the microenvironment of the lymph node where immune recognition is initiated. Antigen-presenting cells engulf and transport microbes from the site of infection *via* the lymphatic vessels to the draining lymph nodes where they present the constituents of the microbe to the patrolling B- and T-cells. In this way, the concept of a microbe can be formed during an infection. However, in the context of a vaccination, when only parts of a microbe, i.e., single antigens or even epitopes are used as a vaccine, the concept of a microbe cannot be formed. So when, e.g., only the toxin of a bacterium is used for vaccination, the pathogenic toxic effects of an infection might be prevented, but infection as such still occurs, because the immune system has no concept of the bacterium.

### Dynamic Aspects of Concept Formation and Immunodominance

The formation of a concept by the immune system is not a one-step procedure, but a continuous process. In the first encounter of a naïve B-cell population with a new antigen in a primary immune response, the whole surface of an antigen might serve as a collection of potential-binding sites. Such early binding, low-affinity B-cells are, however, soon replaced, through a selection process, by cells expressing receptors that allow thermodynamically more favorable binding to particular epitopes, depending on the chemical composition of the latter. By a continuous process of somatic receptor mutation and selection of the best-fitting B-cell clones, the affinity stepwise increases and regions in antigenic domains are focused around immunodominant (prototypic) epitopes (16, 17).

### Original Antigenic Sin and Non-Monotonic Formation of Concepts

A characteristic of the immune system is the phenomenon that the immune response often sticks to its old concepts. Re-exposure to a variant strain of virus boosts the response to the original virus that has induced a previous immune response, i.e., formed an original concept. Consequently, responses to new epitopes of the virus are impaired. This phenomenon is called *original antigenic sin* and it poses a problem, e.g., in the development of vaccines to highly variable viruses like influenza virus or HIV. Recently, a mathematical model based on the antigenic distance hypothesis has been developed that predicts with impressive accuracy the ratio between the effect of a repeat vaccination and the primary vaccination against influenza (12). In essence, the antigenic distance between the first vaccine and the second vaccine has to be large enough for the immune system to recognize the second vaccine as a new concept. Generally expressed, there is a non-monotonic change from one concept to another with a certain threshold level of antigenic distance necessary to switch from one concept to the other.

### Learning

Despite the occasional antigenic sin, the immune system is able to learn and to adapt its view on the microbial world. Learning by the adaptive immune system corresponds to expanding the conceptual space with new dimensions (epitopes) or to adapt the salience of a dimension or domain (epitopes, antigen). At an early time point of an acute infection with a particular microbe, usually a different set of antigens and epitopes are salient for the immune system than at a time when infection is resolved or is getting chronic or latent (18). This change of perspective by the immune system is important to analyze when diagnostic tests are used to diagnose an infection and to follow the course of disease by measuring the immune response to the infecting agent. Characteristic patterns of reactivity to particular antigens or epitopes are often instrumental in distinguishing various stages of an infection.

But more than that, immunological *experience* gained by encountering a particular microbe might well affect the way a different microbe is recognized, particularly when the new agent *looks* similar to the old one (19, 20).

## Context and Genetic Effects on the Salience of Domains

The strength of an immune response to a particular epitope, i.e., the immunogenicity of that epitope, can be measured by the number of lymphocytes responding to it. In the framework of conceptual spaces that measure can be represented by the salience of an epitope dimension.

An important characteristic of an immune response is the cooperative effect of T-cells and B-cells. B-cell receptors recognize certain epitopes of an antigen (B-epitopes), then the antigen is ingested and digested by the B-cell and other epitopes of the same antigen are presented to T-cells (T-epitopes), which when responding give help to the B-cells. Consequently, the salience of a B-epitope is strongly dependent on the presence or salience of T-epitopes. In this way, the immunogenicity of B-epitopes is strongly context dependent.

### Genetic Regulation of Antigen Presentation

Immune responses are strongly regulated by immune response genes. Most importantly, the highly polymorphic MHC genes, which play a major role in immune regulation, code for antigen-presenting molecules on the surface of antigen-presenting cells. Like that, in every individual organism, the dimensions of epitopes have different weights. Some epitopes might not be presented at all in the context of a particular MHC type, thus making the immune system *blind* for that dimension.

## Vaccines as Metaphors

The hypothesis here is that vaccines are metaphors of microbes. It is based on the view of Gärdenfors’ that

a metaphore expresses an identity in topological or geometric structure between different domains.

An antigen in a vaccine expresses the same structure as an antigen in a particular microbe, but it is not part of the same infectious microbe. A vaccine is either an attenuated microbe (as in live vaccines) or a dead microbe or it is a pure antigen not attached to a live or dead microbe. The metaphoric nature of a vaccine is particularly obvious when single epitopes that make up the property of an antigen are artificially expressed in a viral vector (like vaccinia virus) or are used as a mixture of single peptides.

Sometimes, the *meaning* of an antigen (see Semantics of the Immune System) that is applied as a vaccine is lost or distorted by the context of the vaccine, and consequently, the immune response elicited by the vaccine might not be protective.

### Combining Concepts by Vaccine Construction and in Viral Infection

A particular interesting question in applying the framework of conceptual spaces to immune cognition is the combination of concepts. Gärdenfors’ formulates the following rule for combining concepts:

The combination CD of two concepts C and D is determined by letting the regions for the domains of C, confined to the contrast class defined by D, replace the values of the corresponding regions for D [(7), p. 122].

To take an example from the immune system, one could envisage the construction of a vaccine by genetically engineering an antigen into a vector virus, i.e., combining an antigenic property with a concept. Like that, the immune system recognizes the antigen in the context of the vector virus (contrast class) and the vector virus exhibits a new antigenic property. The prediction within the framework of conceptual spaces would be that the saliences of the inserted epitopes are influenced by the type of vector virus used and that the immunogenicity of the vector virus is dependent on the new antigenic property. Such predictions are amenable to experiments.

Another example of combining concepts would be the insertion of a viral concept into the concept of self when the virus is infecting cells and the infected cells are consequently expressing viral antigens. Like that, a foreign property is combined with self. Whether such newly formed concept of virus-modified self will lead to the destruction of the infected cell or to tolerance of the new concept is a matter of the semantic *interpretation* by the immune system (see Semantics of the Immune System).

A particularly interesting case is given when a well-tolerated concept of virus plus self is suddenly challenged by the viral concept presented in a semantically new context (21). In an experimental model of a transgenic mouse, carrying viral antigens in the insulin-producing β-cells of the pancreas, tolerance to the viral antigens is broken when the mouse is infected with the same virus. Consequently, the viral antigen carrying β-cells are destroyed by the immune system and diabetes develops. Cognition of the foreign concept (virus) changes the self-concept (host cells).

## Immune Escape by Camouflage

One way in which an infectious agent can evade immune *surveillance* is by altering its antigenic property. Well-known examples are the recurrent epidemics of influenza. The virus has developed two strategies that allow evading neutralization by antibodies. The milder form, called antigenic drift is caused by point mutations in the genes encoding surface antigens of the virus. In this way, the saliences of the epitopes are changed and although the virus still looks similar for the immune system, the defense is less vigorous and infection prolonged. Major influenza pandemics result when the second strategy comes into play, the antigenic shifts caused by re-assortment of the genome of the influenza virus and related animal influenza viruses in an animal host. The resulting virus is not recognized anymore by the immune system knowing the concept of the previous variant but being ignorant to the new antigens disguising the virus. Consequently, severe and sometimes fatal infections result. In a worldwide endeavor, each year new vaccines are constructed, which contain the new antigenic variants of the virus and that allow the immune system to learn in advance the new viral concept.

## Semantics of the Immune System

The semantic interpretation of a recognized phenomenon is a key feature of a cognitive system and for biological cognitive systems it is very likely central to survival. This tenet holds also for the immune system.

The hypothesis is that the *meaning* of an antigenic stimulus, in form of a signal to an immunological receptor, is given by the response of the cell carrying that receptor. Cohen writes: “Indeed, the nature of the response – its quality, quantity, timing and location – is what gives effective meaning to the recognition” [(5), p. 442]. The stimulus is *interpreted* by coupling of the receptor to intracellular signal pathways that induce cellular responses appropriate to the stimulus. Here, it is important that we distinguish between the innate immune system and the adaptive immune system. In the innate immune system, the specificities of the receptors are genetically encoded, whereas the antigen receptors of the adaptive immune system and their specificities are generated by random processes. The latter are the antigen-specific T- and B-cell receptors. Such random specificities of the receptors are clonally distributed, i.e., each clone of T- and B-cells carries a particular, but random, specificity. Medzhitov and Janeway distinguish non-clonal germline-encoded receptors of the innate immune system and clonotypic receptors of the adaptive immune system (22). The authors argue that the semantic information is conveyed by the non-clonal recognition system, because randomly created receptors cannot carry semantic content as they would not *know* in advance what antigen they will recognize and what type of response they will have to induce. In Cohen’s words: “The naked epitope cannot tell an inexperienced lymphocyte which type of response is appropriate; information about the context is necessary” [(5), p. 443]. Key components of the innate immune system for conveying semantic information are the dendritic cells with their toll-like receptors that recognize pathogen-associated molecular patterns.

### Context Influence on Semantics

The actual immunogenicity of an epitope as expressed by the number of lymphocytes responding to the epitope (salience of a dimension) depends on its context.

Zinkernagel et al. expressed this context dependence by a “geographical view of the immune system” (23, 24) and concludes:

Collectively the data indicate that antigen, dependent upon localisation, dose and time, seems to be the simplest regulator of immune responses.

Borghans et al. describe the context dependence in the following way:

We adopt the view that the innate immune system provides signals about the context of antigenic epitopes. Depending on 1) the organ where the epitope is detected, 2) the presence of conserved pathogen-associated molecular patterns, and perhaps 3) tissue damage, the innate system signals whether the Ag should be attacked and if so, by which immune effector mechanisms (25).

Changing the contexts might be one of the mechanisms of action by immunotherapeuticals like cytokines or antibodies against them or their receptors.

### Autoimmunity as a Semantic Function of the Immune System

Autoimmunity is a reaction of the immune system toward self that might lead to disease. It is a puzzling observation that B-cells as well as T-cells specific for autoantigens circulate in the healthy organism without doing any harm. It is assumed that these autoreactive cells are somehow functionally downregulated by so far ill-defined mechanisms. That is, from a cognitive point of view, the immune system is not *blind* to autoantigens, in other words, properties in the domain of self are recognized, but the meaning of self-recognition is different from recognition of non-self. According to Medzhitov’s hypothesis mentioned above, it is the innate immune system that is malfunctioning in autoimmune disease by not providing the correct semantics to autoreactive elements of the immune system. The reason for it is still unclear, but one explanation could be molecular mimicry.

### The Learnability of Meaning

How then can the adaptive immune system learn the meaning of a newly encountered antigen? This question is closely related to the question of self/non-self discrimination. Medzhitov and Janeway argue that the innate immune system is instrumental in *instructing* the adaptive immune system with semantic information, and they describe the mechanisms which fulfill this task (26). As such, preexisting germline-encoded effector mechanisms of the innate immune system are enriched with the adaptive and highly specific recognition of any potential antigenic structure.

Medzhitov and Janeway see this as a fundamental principle that might be valid not only within the immune system:

Finally, we suggest that in a fundamental way, the same principles apply to the information gained about “unpredicted” external stimuli in the functioning of the adaptive component of the nervous system [(22), p. 213].

## Conclusion

The representation of cognitive phenomena of the immune system within the theoretical framework of conceptual spaces enables the analysis of immune cognition on a meta-level and the formulation of predictions about cognitive functions that can be tested experimentally without reverting to metaphoric comparisons with the neuro-psychological system. Still, when such analogies to neuro-psychological phenomena are real, a common cognitive theory, like the framework of conceptual spaces, is instrumental in expressing such analogies. On the other hand, differences between two cognitive fields can be formulated on a meta-level that is pertinent for either of them.

Constraints derived from treating the immune system within a cognitive theory, like Gärdenfors’ conceptual spaces, might well prove to be instrumental for the design of vaccines, immunological diagnostic tests, and immunotherapy.

Vaccine construction is an example of combining concepts, e.g., by engineering antigens (properties) into a vector virus or using ISCOMS (immune-stimulating complexes) as adjuvants and thereby changing the saliences of the inserted epitopes by the semantics of immune responses against the viral components used as vector or adjuvant. However, even when immunization to an antigen (property) used in the vaccine is successful the immune system might not recognize the whole concept of the microbe carrying that antigen. Consequently, vaccine construction not only has to consider an efficient way of combining concepts but also assure that the target concept (microbe) to be vaccinated against is presented in an integral form, e.g., as an attenuated virus. Furthermore, when designing vaccines to new variants of a virus, the antigenic distance between the first vaccine and the second vaccine has to be large enough for the immune system to recognize the second vaccine as a new concept.

Diagnostic tests for antibodies to microbial antigens should be based on prototypes of antigens (natural properties) that are encompassing a large enough variability of properties of the individual immune systems in the population. Furthermore, in order to distinguish different stages of an infection, diagnostic tests should distinguish different concepts of the infectious agent that the immune system develops during the course of an infection. A concept and the affinities of the epitope recognition by the immune system at the beginning of a fresh infection might well be different from a concept and the corresponding affinities after successful elimination of the microbe or during lifelong latent persistence of the microbe.

Immunotherapy with the so-called biologicals like cytokines or antibodies against them or their receptors influences the semantics of immune responses by changing the context and thereby the saliences of immune reactions in certain antigenic domains. T-cell-based cancer immunotherapy, as another example, requires the identification of ideal cancer antigens, i.e., natural properties, as well as achievement of the right immunological semantics to enhance *in vivo* persistency and survival of adoptively transferred T cells (27).

In addition, cognitive functions of the immune system, which are well embedded in a theoretical framework, can inspire the fast growing field of AIS (28).

## Author Contributions

The author confirms being the sole contributor of this work and approved it for publication.

## Conflict of Interest Statement

The author declares that the research was conducted in the absence of any commercial or financial relationships that could be construed as a potential conflict of interest.

## Footnotes

^1^Terms used within the context of the immune system, which are related to or borrowed from the neuro-psychological cognitive context, are written in *italic* font on their first encounter.

^2^Terms defined within the framework of conceptual spaces are written in monospace font.
